# Medical News and Miscellany

**Published:** 1887-02

**Authors:** 


### MEDICAL NEWS AND MISCELLANY.

A BILL TO CREATE Local Boards of Health in unorganized towns and counties has been presented by Senator (Dr.) Camp, of Limestone, and it is thought it will pass. These local boards are to be subordinate to the State Health Officer.

CHOLERA.—The health authorities of New Orleans are very active in the endeavor to prevent the introduction of cholera from the Argentine Republic, vessels arriving from that country being immediately sent to quarantine. The cholera has reached Chili and is creating havoc.

SNOWED UNDER AGAIN.—We learn from a Senator (a doctor) that the bill to create a State Board of Health for Texas, presented by the State Medical Association's Committee on Medical Legislation, was "killed in the Senate committee." The committee consists of Drs. Geo. Cupples, J. W. McLaughlin, T. D. Wooten, T. J. Tyner, Clopton, Pope and Kelley. The pharmacy bill, presented by the State Pharmaceutical Association, also failed. No effort has been made to secure the passage of "a bill to regulate the practice."

CINCHO QUININE has all the advantages of quinine, without its disagreable taste, which, in the cases of children, is a point of much importance.

---

THE AMENDE.—In commenting on Dr. Osborn's paper in our last, we said that Dr. Osborn claims that measles and dengue are non-contagious, or words to that effect. The Doctor specified "Rotheln" or German measles, and "Roselina" or Italian measles as being non-contagious, and not Rubeola.

---

THE PHARMACY BILL.—Just as we expected the Senate Committee on Public Health and Vital Statistics (consisting mostly of doctors) to whom was referred the very excellent and much needed bill to regulate the practice of pharmacy in Texas, presented by the State Pharmacal Association, reported adversely, and the bill failed to pass.

---

DR. W. H. THOMPSON was killed at Axtell, McLennan county, Texas, on the 15th inst. by two men named Pippin, father and son. There was a horse race in progress and Thompson, who was said to be in liquor, got into a difficulty with one Sneed, a brother-in-law of Pippin, when younger Pippin shot him down, and the elder clubbed him with a pistol. The Pippins have fled. We know nothing of Dr. Thompson.

---

HARDLY FAIR.—Our friend Gray of *Atlanta Medical and Surgical Journal*, in a card to advertisers, claims that that publication is the only strictly first class medical journal in the South. This is the first intimation we have had that ours is considered a Western Journal. Now, in what class does brother Gray place the *New Orleans Medical and Surgical Journal*, and the *Mississippi Valley Medical Monthly* and several other high-toned, able and courteous Southern contemporaries? What constitutes "first class" that is not to be found in them? The *Atlanta Journal is* first class but not the only one.

---

SHIP ISLAND QUARANTINE.—Surgeon General Hamilton, M. H. S., advises the removal of the quarantine from Ship Island to Grand Crozier Island one of the Chandeleur group in the vicinity, and has submitted to Congress an estimate of the cost of removal—a total of $45,000. In accordance with the above, bills have been introduced by the senators from Mississippi and Louisiana, providing for a transfer to "some other island in the Gulf of Mexico or the Mississippi delta; the site to be selected by a board to be designated by the Secretary of the Treasury." The bill also appropriates the amount recommended in the estimate, "or so much thereof as may be necessary."

---

THE REPORT ON TEXAS SURGERY.—This valuable compilation by the Special Committee appointed by the State Medical Association, of which committee Dr. Geo. Cupples is chairman, is now ready for delivery to members. It is, properly, an appendix to the transactions, but was, necessarily, published in separate form. Every member is entitled to a copy, but as the Secretary has no funds belonging to the association, with which to pay postage, and presuming that members will be willing to pay the postage in order to get a copy, rather than wait till the April meeting, the Secretary now gives notice that a copy will be sent to all members who will enclose to him the necessary postage—seven cents each copy. There are 600 copies in the hands of the Secretary for distribution.

---

AN UNJUST IMPUTATION.—In Dr. Briggs' article he says, in speaking of his "Prize Essay" in the "Transactions": "There may be lack of commas and semi-colons; and colons and periods may have been incongruously mixed; in fact the make-up of the Essay, by the printers, may be inartistic in the extreme," etc. We can testify that the "Prize Essay" was printed as written, with the exception of certain corrections, (errors of grammar and clerical errors) which we made, at the Doctor's earnest solicitation. The "make-up" of the article "by the printers" was *not* inartistic, but was an exact reproduction of the article, read by copy, by a competent proof-reader before it came to the publishing committee, by whom it was read also, and read a second time by both, from revised proof, and commas and periods were not "incongruously mixed."

---

ERRATA.—In the January number of this JOURNAL, in the editorial "Baking Powders and the Public Health" we inadvertently said "elixir vitriol," whereas we meant to say "oil of vitriol." We expect to see a dozen or so small fry pitch into us for it, but can't help it. Also in Dr. Osborn's excellent paper, on the "Intimate Connection Between Areas of Low Barometer and the Prevalence of Epidemics of Non-Contagious Diseases," there occurred a single typographical error—on page 269—sixth line in paragraph on the means for the seven months of '85—for $22.^{29}$ read 22.29. There were one or two minor typographical errors on page 294 in "Medical News and Miscellany," which were seen and marked. but the corrections were not made in the revision of the proof, to-wit: "gynaœcology" should be "gynæcology," "Corriz Springs" should be "Corrizo Springs" and "Quinism" should be "Quininism." Accidents will happen in the best regulated families they say, and printing offices are no exception to the rule.

---

PERSONAL.

Dr. A. N. Denton, late Superintendent State Lunatic Asylum, has located for practice in Austin.

Dr. W. H. Wilkes, late of Waco, Texas, now Lecturer on Diseases of Children in the Kansas City, Mo., Medical College, and associated with the Drs. Jackson, in practice, has accepted a professorship, and next session will fill the chair of Theory and Practice, or of Obstetrics; either one of which he is competent to grace. It will be gratifying to his large number of friends throughout Texas to learn that the Doctor will attend the next meeting of our State Association; if he should do so, he may consider himself "booked" for one of those able and graceful speeches for which he is so justly noted. Welcome, most cordially.

---

A Brilliant Hit.—The story of the doctor who assured the parturient patient that he "understood her case," and that he "had a man patient up town affected in the same way," has been discounted lately by a Texas physician, not a thousand miles from Austin, who made a diagnosis off hand with great confidence. He was consulted by the wife of an influential citizen, who said, "Doctor, my baby is strangely affected, and I want you to tell me what's the matter. Whenever it goes to make water it seems to suffer great pain, and almost goes into convulsions, turning blue in the face, and throwing itself back till nothing but the heels and head touch the bed. What is the matter with my baby?" "Why, my dear madam," replied the doctor, with the pompous and confident air of one who saw his opportunity for a brilliant hit, "it is a clear case of elongated, and perhaps adherent prepuce." "Ah! indeed," said the lady; "and are you *sure* Doctor that is what ails it?" "Perfectly sure madam, couldn't be mistaken; had thousands of such cases." "And what can you do to relieve it, Doctor?" "Oh, simple enough my dear madam," said the doctor, "I'll just remove the surplus prepucial tissue, stitch the integument and the mucous membrane back with a couple of antiseptic sutures, and the little fellow will be all right," said the doctor, with a bland and know-it-all smile. "But," said the lady, with a mischievous twinkle in her eye, "Doctor, my baby is a girl!" Exit the doctor, who had a patient to see, just about that time. Fact.

---

THE "PRIZE ESSAY" AGAIN.—It was announced in our last issue that Dr. Briggs (to whom the prize of $100, offered by the Texas State Medical Association for the best original paper, had been awarded by a majority of the committee) had accepted Dr Wallace's challenge, to-wit: that the paper be submitted to three recognized psychologists, one to be chosen by each Dr. Wallace and Dr. Briggs, and one by the President of the Texas State Medical Association, and if they decide the paper to be a prize paper, Dr. Wallace agreed to pay Dr. Briggs $100 additional out of his own pocket; if not, Dr. Briggs was to return the $100 to the treasury of the Texas State Medical Association.

In making the announcement we supposed, naturally, that Dr. Briggs understood the very obvious meaning and intention of the offer' and had accepted it in good faith. He announced his readiness to accept the wager, and offered to put up $50 forfeit,and challenged Dr. Wallace to do the same. Now it transpires in a correspondence between the gentlemen that Dr. Briggs takes Dr. Wallace up on a technicality, and claims that the referees selected have nothing to do with the *merits* of the paper, but are to decide simply whether his paper, having been pronounced and published as a Prize Essay, *is* a Prize Essay. Techically, there could be no question on this point, and we have heard it said that—even amongst sporting men—a mere catch or techicality, or in the vernacular, "a *dead sure thing*" does not win. It is very evident to any unbiased reader, that Dr. Wallace's meaning was, whether the paper had sufficient merit to entitle it to the prize, the committee of award having differed on the subject, and the award having been made by three out of the five. Thus while Dr. Briggs has ostensibly met the issue,he has practically evaded it, and we have to retract our statement in last issue. We venture the opinion that the profession will not sustain Dr. Briggs in a course which has, to us, much the appearance of a subterfuge. The end is not yet.

---

THE CHANGES AT THE LUNATIC ASYLUM.—In our last issue we asserted that no change would be made in the medical staff by Dr. Dorsett, the incoming Superintendent at the Lunatic Asylum. As a clean sweep of every attendant, medical and other, was instantly made upon the Doctor's taking charge, we can only say that he, himself, was our authority for the statement. Before his confirmation Dr. Dorsett gave us his positive assurance that he would "make no changes, at least for the present," and authorized us to assure Drs. Bennett and Maxwell, the two assistant physicians, that they would be retained. As Drs. Preston, of Seguin, and Davis, of —— were appointed to supercede them, and claim as we learn, that they were here under assurance of appointment at the time Dr. Dorsett gave the above promise, it remains for Dr. Dorsett to explain this seeming breach of faith, which no doubt he can do satisfactory. Of the new appointees we know nothing.

---

OH! WHERE SHALL REST BE FOUND?—Please give us a rest on "Don'ts for the Sick Room." If there ever was a "chestnut" that is one of the sickliest sort. We honestly believe that this is the only JOURNAL in the United States that has not copied the abominable thing. We furnish our own paragraphs; and if our esteemed contemporaries will keep their eagle eye on the RED BACK JOURNAL they will always find something fresh. A friend remarked, "When I take one journal I take all, for I see the same thing in all of them." No wonder, when such things as "Doctoring an African King," "He Snatched Her from the Grave" and "Don'ts for the Sick Room" go the rounds till they are threadbare. Give us a rest. Now, after eight months, here comes the *Lancet and Clinic* and accuses "a Texas Medical Journal" of *saying* "when the baby is done drinking it should be unscrewed and hung up." We got that off on some other fellow, last year, and it has gone the rounds till it is quite out of shape, and comes back at us like a boomerang. We have a good one this month; two of them, in fact, and as we launch them we have an abiding faith that we shall see them again and again, and perhaps, have them hurled back at us.

---

HE SAID IT.—When was our friend Dr. F. R. Martin of Kyle, Texas, presumably in luck? When he first struck Kyle!—Oh!

---

We sent a "sample copy" by request, to a Doctor Joseph Smite, and Dr. Merryman wrote to inquire what, if any, relation he is to the widow Smite, spoken of in the Bible.

---

WANTED.—Position in Drug Store, by a No. 1. prescriptionist, thoroughly experienced; single man; best testimonials to character, habits and capacity. Address R. P., care Editor of this JOURNAL.

---

HARD UP.—"Our esteemed" of the *Medical Record*, and of the *Courier Record*, must be hard up for a pretext to hit us, when tbey can find nothing to criticise but a *purely typographical error*, one so clearly typographical that any but unfriendly eyes could see it and understand at least what was meant, (see "early voting" for "early vomiting.")

---

NATIONAL ADULTERATION BILL.—The National Board of Trade has submitted to Congress, and are urging its passage, a bill, defining the offense of adulterating food or drugs, and providing a penalty therefor. It is very sweeping, and, in case of drugs, embraces articles differing in "strength, quality or purity, from the standard of the U. S. P;" and in case of food or drink, an article is said to be adulterated, not only when it has any substance mixed with it to lower its quality, but if it consists in whole or in part of "decayed or diseased or putrid or rotten animal or vegetable substance;" and milk from diseased animals comes under the head of adulterated food. The bill is designed to prohibit the sale of every form of food deleterious to public health.

---

A COMEDY OF ERRORS.—The *Texas Courier Record* falls into quite a number of errors; for instance, it deprecates Dr. Swearingen's "removal" from the office of State Health Officer. Dr. Swearingen was no more "removed" than Gov. Ireland was.

He had served *three full terms*, and his last term expired with the incoming of the new administration. He was simply not re-appoinfed.

Again, the C. R. *condoles* with Dr. Ghent, who has been "made a co-respondent in a suit brought by Dr. M. Salm, late of Austin," [for damages] and says: "We predict that Dr. Ghent will not be found wanting in presenting a vigorous and successful defense against the heinous crime [sic] with which he is charged"—[! ! !] This is as startling as it is unfounded, Dr. Ghent is not charged with any "heinous crime"; he is simply being sued for damages by one whose "heinous crime" he exposed.

---

HOW CHOLERA WAS CARRIED TO BUENOS AYRES IS SOUTH AMERICA.—In the *Weekly Abstract of Sanitary Reports* (No. 48) dated January 27th, issued by the Treasury Department, M. H. Service, the following occurs:

"BUENOS AYRES.—The United States Minister, under date of December 3, '86, dispatches that the situation is not alarming at present, but says:

'There is no room for doubt as to the existence of Asiatic cholera here. It made its first appearance about five weeks ago, and was imported by the Italian ship, Perseo, plying between Genoa and Buenos Ayres. Dr. Antonio del Veso, envoy extraordinary, and minister plenipotentiary of the Argentine government in Italy, was a passenger on the ship, and the anxiety to secure him an immediate landing, on the part of the ship's commander seems to so far overcome his sense of duty, that by concealed or garbled reports, he managed to turn loose, on Argentine soil, first here, then at Rosario, a great many persons from an infected ship. The testimony of passengers shows conclusively there was nearly a score of burials at sea of those who died of cholera on the voyage.'"

The U. S. Minister does not say what was done with the cowardly fellow, who, to save his own worthless life, sacrificed thousands of others—and the end is not yet. What punishment should such a person—a high government official—receive?

---

THE NEW STATE HEALTH OFFICER; OTHER APPOINTMENTS.

DR. R. RUTHERFORD of Houston, has been appointed by Gov. Ross, to be State Health Offieer, vice Dr. R. M. Swearengin, whose term of office expired January 1st. On the 29th January, the Senate confirmed the appointment by the requisite two-thirds vote, and the Doctor enters at once upon the discharge of the important and responsible duties of the office. We believe Dr. R. was the first State Health Officer of Texas; he having been appointed by Gov. Roberts during his first term, shortly after the passage of the bill creating the system. Gov. Roberts appointed Dr. Swearengin during his second term of office; and Gov. Ireland continued him in the position during his two terms as Governor. Dr. Rutherford has many warm friends who are exultant over his appointment, and the public confidently look to see him so administer the quarantine system as to keep up the reputation of the office to the high standard maintained during the terms of service of his predecessor. We wish him success.

Dr. T. J. McFarland of Indianola, who, it will be remembered, lost all his worldly goods in the storm, in August last, and who, with his family sought safety on the house tops—whence they were rescued—has been appointed Quarantine Officer at Pass Cavallo, below Indianola. This position has been filled by Dr. F. K. Fisher heretofore, but the Doctor, who suffered equally with Dr. McF., wanted "no more of it in his," and has gone to Galveston to practice. We congratulate the new Health Officer, and the Governor in the appointment of so worthy and so capable an officer as Dr. McFarland.

Dr. D. R. Wallace, Superintendent Texas Lunatic Asylum.—We are pleased to learn that this efficient and faithful officer is to retain his position, not withstanding the general sweep of removals. It was rumored lately that he, too, would be asked to step down and out.

Dr. T. C. Thompson, of Galveston, (Druggist) has been appointed on the Board of Regents to fill a vacancy caused by the death of Dr. Ashbell Smith, President. Dr. Wooten having been elected to the presidency some time since.

Dr. Preston, of Seguin, has been appointed First Assistant Physician at the State Lunatic Asylum, vice Dr. T. J. Bennet, (who resumes practice in Austin) and Doctor Davis, late of Gatesville, second assistent, vice Dr. F. A. Maxwell. Mr. J. Kendall, of Throckmorton, has succeeded Dr. Shapard in charge of the Institute for the deaf mutes.

A Medical College in Texas.—The following was adopted by the North Texas Medical Association at its last meeting, held in Paris, Texas, in January (ult.)

Whereas, Provision has been made for the establishing of a medical department in connection with the State University and no steps have been taken to put it in successful operation, and whereas inferior medical colleges are springing up all over the country and are liable, also, to be established in this State, in order to counteract such a step it devolves upon the medical profession in its organized capacity to take an active part in the establishment of a thoroughly first-class, scientific school of medicine in this State for the sake of humanity and the honor of the profession. Therefore be it

*Resolved*, first, That it is the unanimous sentiment of the members of the North Texas Medical Association that the time has arrived and is now pressing upon us as a necessity when the medical men of the State should put forth a determined and united effort to establish and put in successful operation at as early a time as practicable a first-class medical college in our State.

*Resolved*, second, That we, the members of this association, now numbering one hundred and twenty-five graduates of medicine, pledge ourselves to the hearty co-operation with our brethren of the state in any feasible plan which may be inaugurated to accomplish this desirable end.

Apropos, we clip the following from the *Galveston News* special to the *Statesman* of the 14th inst.

TEXAS MEDICAL COLLEGE.

*Movement to Get the Institution in Running Order in Galveston.*

Galveston, February 14.—At a special meeting of the city council, this evening, it was voted unanimously, that an ordinance should be introduced and adopted at the next regular meeting, donating to the State for the purpose of erecting a medical college thereon, one-half of the valuable block of ground situated in the east end of the city now occupied by the public school, the other half to be utilized for the Sealy Hospital, the erection of which is provided for by the magnificent donation of $50,000 from the estate of the late John Sealy. The mayor has appointed a committee to go to Austin and labor with the legislature on the matter of appropriating money to carry on the medical college.

And as bearing on the subject the following, from Dr. T. D. Wooten, President Board Regents Texas University, to the *Statesman* of 16th inst. will be of interest.

* * * "The University has an income, derived mainly from its bonds amounting to $44,000. The annual expenses on $44,-

000[?] in case the lands are placed under the management of the regents, it is believed that in one year at least one million acres can be rented at 4 cents per acre net. This will produce at the end of the two years, $40,000 [?] It may be possible at the end of the year, to lease the second million acres; if so, at the end of the fourth year the University will have an accumulated available fund of $160,000. At the end of the fifth year, from the present time, or in 1892, $240,000.

Out of this amount the regents will then be able to set aside the following:

| | |
|---|---|
| For completing main building | $180,000 |
| For completing mess halls | 20,000 |
| Additional professors in academic department | 30,000 |
| Balance | 10,000 |
| | $240,000 |

At end of sixth year, deducting the $30,000 for increase in the academic department, and adding the above balance, there will be on hand from loans, etc., the sum of $60,000; at end of seventh year, $110,000; at end of eighth year, $160,000. Then the regents could set aside $140,000 for medical buildings at Galveston, with $20,000 as running expenses for the first year. The next year, or nine years from now, and every year thereafter, $40,000 could be given to Galveston which would make the medical school there one of the finest in the country. After the ninth year, or in 1896, there would be about $10,000 over and above the amounts needed in Austin and Galveston."

